# Empowering precise advertising with Fed-GANCC: A novel federated learning approach leveraging Generative Adversarial Networks and group clustering

**DOI:** 10.1371/journal.pone.0298261

**Published:** 2024-04-10

**Authors:** Caiyu Su, Jinri Wei, Yuan Lei, Hongkun Xuan, Jiahui Li

**Affiliations:** 1 Guangxi Vocational & Technical Institute of Industry, Nanning, Guangxi, China; 2 Universiti Pendidikan Sultan Idris, Tanjong Malim, Perak, Malaysia; 3 Guangxi University of Foreign Languages, Nanning, Guangxi, China; Anhui University, CANADA

## Abstract

In the realm of targeted advertising, the demand for precision is paramount, and the traditional centralized machine learning paradigm fails to address this necessity effectively. Two critical challenges persist in the current advertising ecosystem: the data privacy concerns leading to isolated data islands and the complexity in handling non-Independent and Identically Distributed (non-IID) data and concept drift due to the specificity and diversity in user behavior data. Current federated learning frameworks struggle to overcome these hurdles satisfactorily. This paper introduces Fed-GANCC, an innovative federated learning framework that synergizes Generative Adversarial Networks (GANs) and Group Clustering. The framework incorporates a user data augmentation algorithm predicated on adversarial generative networks to enrich user behavior data, curtail the impact of non-uniform data distribution, and enhance the applicability of the global machine learning model. Unlike traditional approaches, our framework offers user data augmentation algorithms based on adversarial generative networks, which not only enriches user behavior data but also reduces the challenges posed by non-uniform data distribution, thereby enhancing the applicability of the global machine learning (ML) model. The effectiveness of Fed-GANCC is distinctly showcased through experimental results, outperforming contemporary methods like FED-AVG and FED-SGD in terms of accuracy, loss value, and receiver operating characteristic (ROC) indicators within the same computing time. Experimental results vindicate the effectiveness of Fed-GANCC, revealing substantial enhancements in accuracy, loss value, and receiver operating characteristic (ROC) metrics compared to FED-AVG and FED-SGD given the same computational time. These outcomes underline Fed-GANCC’s exceptional prowess in mitigating issues such as isolated data islands, non-IID data, and concept drift. With its novel approach to addressing the prevailing challenges in targeted advertising such as isolated data islands, non-IID data, and concept drift, the Fed-GANCC framework stands as a benchmark, paving the way for future advancements in federated learning solutions tailored for the advertising domain. The Fed-GANCC framework promises to offer pivotal insights for the future development of efficient and advanced federated learning solutions for targeted advertising.

## Introduction

This paper proposes and investigates a new framework—the Federated Generative Adversarial Network and Clustering Aggregation (Fed-GANCC)—to address the challenges encountered during the rapid growth of the global internet advertising market. The growth rate of the advertising market has increased by approximately 20%, from $250 billion in 2017 to $430 billion in 2021 [1–4]. However, challenges such as privacy leaks, high communication costs, significant data discrepancies, and the formation of isolated data islands, non-identically independently distributed (non-IID) data, and concept drift persist during this growth.

The growth of the global internet advertising market is challenged by the formation of isolated data islands. An isolated data island refers to the situation where data is trapped in silos and is not easily shared or integrated with other data. This phenomenon occurs primarily due to three reasons: user reluctance to share data, strict data sharing policies, and high communication costs. In particular, amidst the growing emphasis on data privacy, a solution that complies with the EU’s General Data Protection Regulation and China’s Internet Personal Information Security Protection Guidelines is necessary [5, 6]. Moreover, even with the widespread implementation of 5G, coping with the substantial user behavior data generated by the 4.9 billion internet users in 2021 [7] and an average weekly online time of 28.5 hours [8] remains a challenge.

To address these challenges, we employ federated learning, Generative Adversarial Networks (GANs), and multi-center federated learning. Federated learning provides a secure, decentralized solution for exchanging user behavior data [9, 10]. However, due to the unique behavior patterns of each user and the unique features of the data stored on their devices, the data distributions on individual user devices may differ from the overall data distribution, resulting in non-IID data distribution in targeted advertising scenarios [11–13]. Hence, we introduce GANs to address non-IID data. However, to better cope with the issue of concept drift, multi-center federated learning is also required.

Therefore, how to speed up the training of the global ML model and improve its accuracy has become a new focal point, which also brings challenges to non-IID data. Another significant challenge in targeted advertising is concept drift. Concept drift occurs when the statistical properties of the target variable, which the model is trying to predict, change over time in unforeseen ways. This can cause previously accurate predictive models to become less accurate as time goes on. Specifically, federated learning aims to accomplish exact ad distribution using the ML model parameters of numerous users. In theory, the parameters of different users should correspond to different global models. However, the shifting behaviors and preferences of users can lead to this drift, making it difficult to maintain a consistent and accurate global ML model. To comprehensively address the challenges of isolated data islands, non-IID data, and concept drift in targeted advertising, this paper proposes the Federated Generative Adversarial Network and Clustering Aggregation (Fed-GANCC) framework. Our approach not only facilitates efficient distributed model computation but also places paramount importance on preserving data privacy.

## Literature review

This section sets out to establish a comprehensive understanding of the existing body of knowledge surrounding the application of machine learning and federated learning in the context of targeted advertising. The ensuing discussion evaluates the methodological approaches, theoretical underpinnings, and practical implications of previous works, illuminating their merits and limitations.

### Machine learning in targeted advertising

Machine learning has surfaced as an instrumental tool in managing multi-party data sharing, an integral part of targeted advertising. Shi and Gu’s work highlighted how using balanced sampling and ensemble learning can improve accuracy while minimizing communication overhead [14]. However, Seker contended that such an approach may not be scalable given the exponential growth of data, proposing instead a predictive model that utilizes feature vectors from advertisement-related data [15]. Kuppusamy extended this line of inquiry by adding a mechanism to adaptively update advertisement recommendations according to user preferences [16]. Furthermore, Malhi et al. leveraged machine learning to tune advertisement deployment parameters, enhancing prediction model accuracy [17].

Despite these advances, challenges persist in improving the accuracy and efficiency of model iterations. Furthermore, the control of user data and the training process by a central server presents potential privacy risks.

### Federated learning in targeted advertising

Federated learning emerges as a viable solution to some of these challenges, providing a decentralized architecture that ensures user autonomy in the training process [18]. Several scholars have devised federated learning frameworks tailored for targeted advertising. For instance, Kumar et al. employed a multi-signal joint approach to create an advertisement data market, improving accuracy while reducing loss [19]. However, its stability is yet to be evaluated under diverse datasets. Jiang et al. utilized a federated sponsorship search auction mechanism, attracting more data providers and facilitating the federated learning ecosystem [20]. Still, Wu et al. pointed out the limited applicability of this approach in predicting native advertisements and proposed a privacy-preserving framework incorporating distributed model and user behavior [21].

Nevertheless, the problem of non-Identically Distributed (non-IID) data remains under-addressed in most federated learning frameworks. The personal biases embedded in user behavior data contribute to a significant disparity in data distribution, resulting in divergences in parameter updates. Furthermore, these frameworks lack mechanisms to mitigate concept drift.

The review of these literatures elucidates the need for a more robust solution to address the pressing challenges in targeted advertising, including data privacy, non-IID data distribution, and concept drift, which this study aims to address.

### The role of federated learning and gans in addressing data heterogeneity and user model bias

In order to address data heterogeneity and reduce bias in user models, many innovative solutions have been proposed in the literature. For instance, the work of Li et al. highlights the use of the Fed-Prox algorithm. This approach extends the Fed-Avg methodology by introducing a regularization term in the user’s local loss function, with the aim to minimize the discrepancy between local and global models [22].

Despite its merits, the improvements offered by Fed-Prox over Fed-Avg are relatively modest. In a response to this, Wang et al. argued that factors such as varying numbers of user’s local updates, different optimization algorithms, and diverse data distributions, could lead to optimization target bias issues, subsequently decreasing the convergence rate of distributed optimization algorithms [23]. To tackle this, they proposed Fed-Nova, an approach that normalizes gradients of each user’s local model before performing a global update, thereby rectifying the optimization target bias.

Turning to specific use cases, Zhu et al. employed Fed-OVA, a method designed for the CIFAR-10 dataset. Their approach involved replacing ten-category classifiers with ten binary classifiers, each dedicated to determining the probability that an image belongs to a certain category [24]. While Fed-OVA effectively mitigates the effects of label distribution bias, it lacks flexibility in cases where a user’s local data contains only one label.

The special issue of concept bias remains largely unaddressed by existing methods. To fill this gap, the Fed-MA algorithm, proposed by Wang et al., leverages model aggregation and aligns the neural structure layer-by-layer [25]. Another novel approach, developed by Yu et al., uses grouped convolution layers to separate features and aggregate them according to the features’ belonging groups, achieved via intra-group weighted averaging [26]. However, it’s worth noting that the Fed-MA algorithm is complex to implement and alters the structure of the original neural network.

To address these complex challenges, Xie et al. introduced a concept known as multi-center federated learning. They implemented the Federated Stochastic Expectation Maximization (Fe-SEM) algorithm in their study [27].

**Fig 1:**
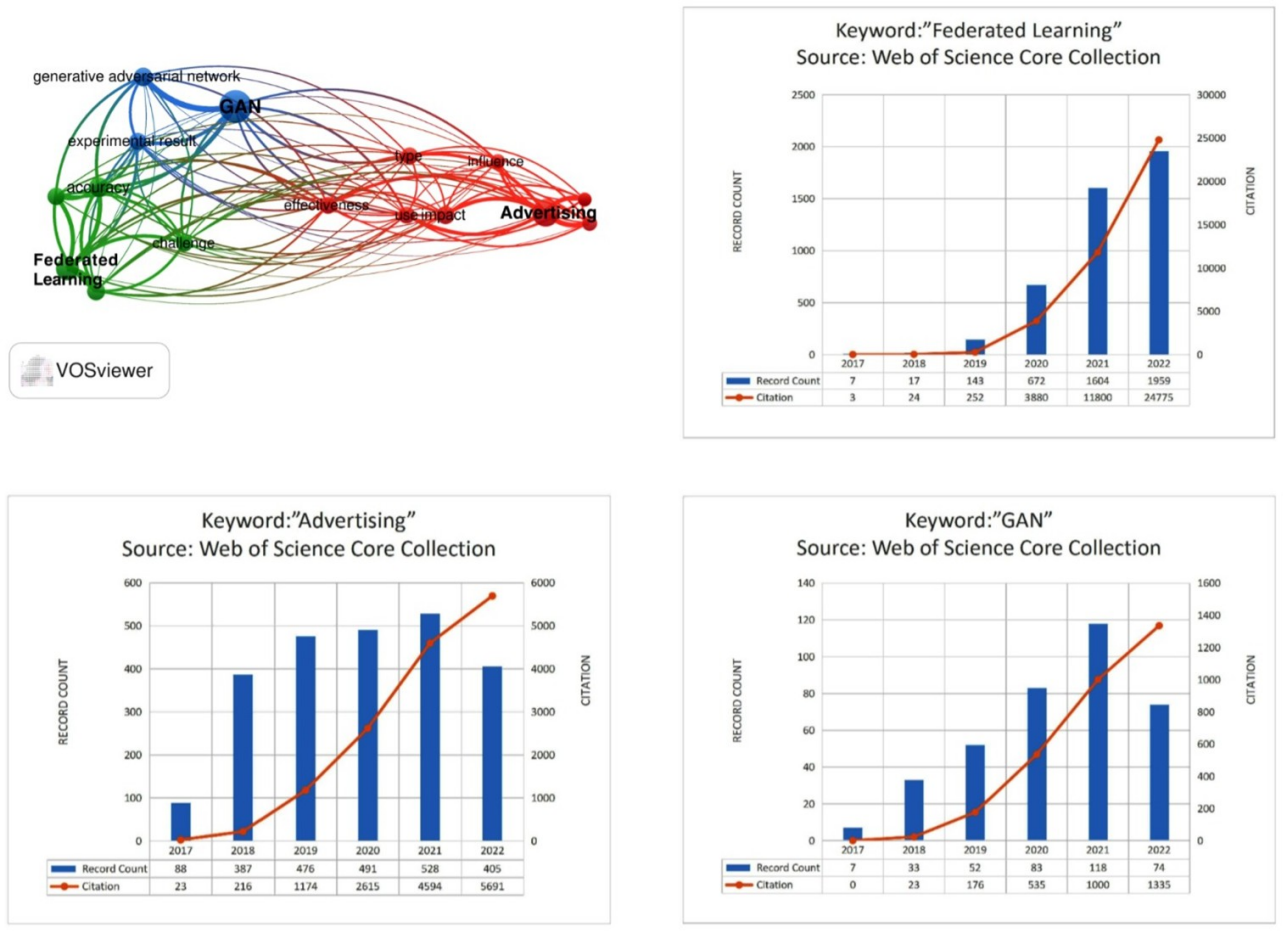
The citation relationship between “Federated Learning”, “GAN” and “Advertising”, as well as the number of publications and citations in the last five years.

Fig 1 visually represents search results from the Web of Science Core Collection for “Federated Learning,” “Advertising,” and “GAN” spanning the last five years. The number of publications for these three topics during this time were 2957, 4595, and 8516, respectively. These numbers suggest an increasing interest in these research areas. However, there appears to be a significant lack of research at the intersection of these areas, indicating a promising direction for future study.

## System model and problem description

### System model

**Fig 2:**
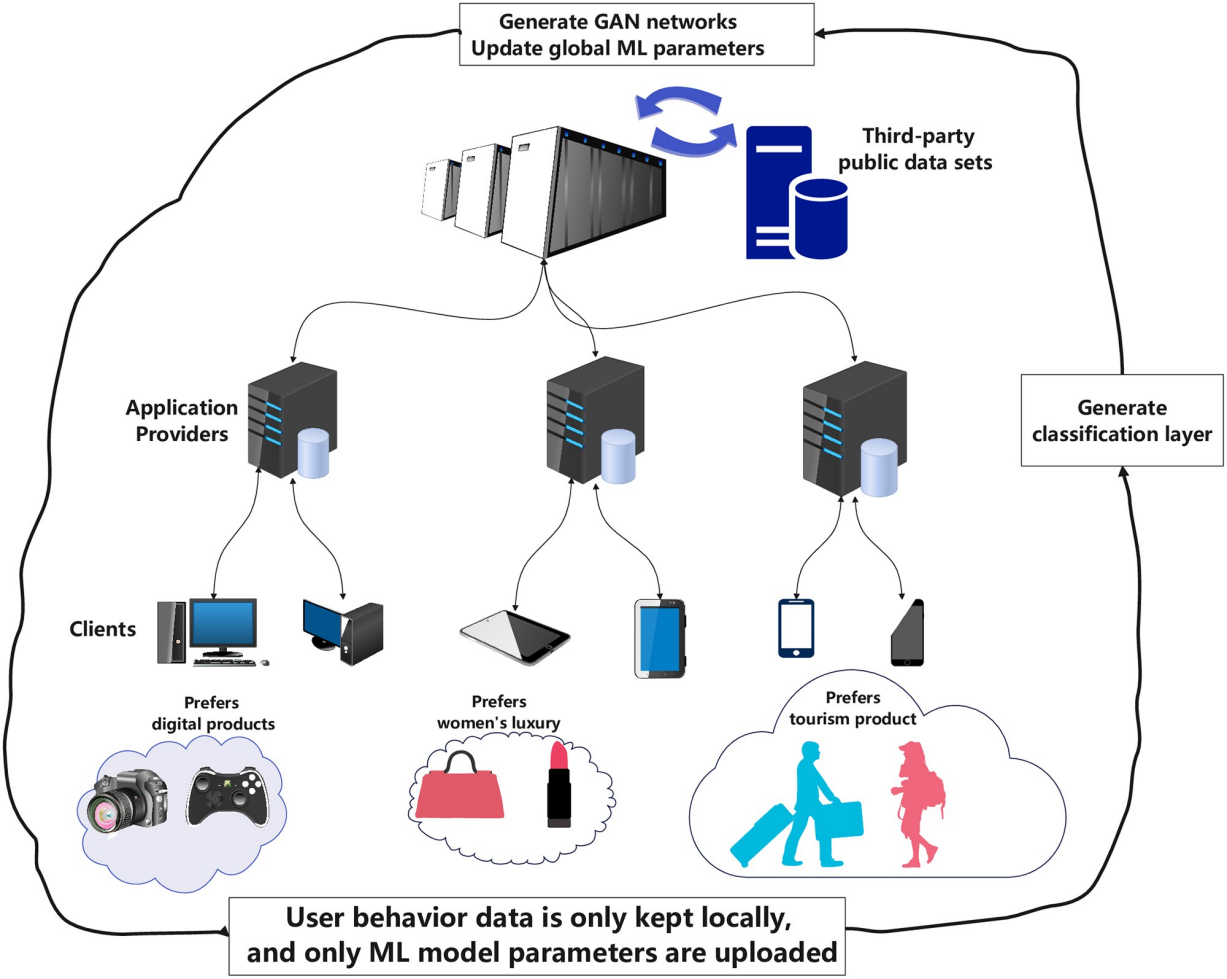
System model.

Fig 2 represents the proposed Fed-GANCC framework. It consists of three core entities: the Central Server, the Application Provider (AP), and the User Node, with the sole transmission of user ML model parameters while ensuring data retention at the user nodes.

- The Central Server: Serving as the key processing unit, the Central Server is furnished only with the parameters of the user’s ML model. It is accountable for strenuous computations such as the generation of adversarial models and execution of personalized group aggregation computations. In our model, we assume that it possesses unlimited computational power and storage capacity.
- The Application Provider (AP): The APs, forming connections with the Central Server, facilitate communication and share the user’s ML model parameters. Their primary responsibilities encompass collecting, uploading model parameters and executing moderately complex computations like creating classification layers based on user’s ML model features. We presuppose that APs possess ample computational power and storage capacity to support these tasks.
- The User Nodes: The user nodes, responsible for data augmentation using the adversarial model, model training and parameter uploading, connect to one AP at a time.

The Fed-GANCC framework excels in enabling personalized advertising without compromising user data privacy. E-commerce platforms, for instance, can use Fed-GANCC to provide tailored product recommendations based on user history, while content providers like streaming services can deliver personalized content suggestions, thereby enhancing user engagement. From a commercial standpoint, Fed-GANCC presents a unique value proposition: by decentralizing data processing and emphasizing model parameter sharing, businesses can improve their advertising strategies while ensuring compliance with data protection regulations. Such an approach can bolster user trust, elevate brand reputation, and potentially offer a competitive advantage. Consider a real-world deployment scenario where an international e-commerce platform aims to offer regional promotions based on diverse user preferences across countries. With varying data protection laws, centralizing user data becomes challenging. Here, Fed-GANCC can shine—regional nodes process user data locally, sharing only model parameters with the central server. This decentralization enables the server to develop a global model that caters to varied user preferences, ensuring effective and regulatory-compliant targeted advertising.

The user-generated data, evolving from web browsing, online services or application usage, resides only at the user node. This gives rise to the user dataset D_*user*_. Given the independent generation of data by each user, the data distribution and volume across devices may not be identical and independent, thereby leading to distinct statistical characteristics that might affect the convergence of algorithms.

To mitigate this, the Central Server initially generates and distributes an adversarial model based on the training dataset. Consequently, users augment local data using this adversarial model. Following this, the enhanced local data D_E_ aids in training the user ML model parameters *θ*_*user*_, which are then stored locally. After the successful upload of *θ*_*user*_, the AP receives these parameters, conducts feature extraction to generate a classification layer and subsequently uploads it to the Central Server.

The Central Server uses the user ML model’s classification layer as a clustering object and applies a clustering algorithm to produce user groups, which are then allocated to their respective user groups. The set of users is defined as U, and a specific user is denoted as U_k_, where k = {1, 2, 3…}. The user U_k_’s sample data is represented as ${\text{D}}_{\text{k}}=\{X_{1}^{\left(k\right)},X_{2}^{\left(k\right)},\ldots ,X_{i}^{\left(k\right)}\}$. The sample data volume of this user U_k_ is denoted as S, and the total training sample volume is $S_{\text{sum}}=\sum_{i=1}^{n}S$.

The symbols used later and their explanations are shown in Table 1.

**Table 1:** Symbols.

Symbols	Explanations	Symbols	Explanations
D_user_	User’s local dataset	$\Gamma_{\text{forget}}^{\left(t\right)}$	Forgotten gate of round *t*
D_E_	User’s enhanced dataset	$\Gamma_{\text{in}}^{\left(t\right)}$	Input gate of round *t*
*θ*_user_	User’s ML model parameters	$\Gamma_{\text{out}}^{\left(t\right)}$	Output gate of round *t*
*θ*_*k*_	ML model parameters for user *k*	$Y_{\text{pred}}^{\left(t\right)}$	Predicted output of round *t*
*θ*	Global ML model	*W*_*k*_	The *k* column of the weight vector *W*
*P*	Total number of users	*C* ^(*t*)^	The state unit of round *t*
*P*_*k*_	The *k*th participating user	$W_{i}^{*}$	Optimal cluster model for the *i*th user group
*S*	User’s sample data	{User}	Collection of all users
*X*_*k*_	Characteristics of the sample data	$r_{k}^{\left(i\right)}$	The cluster belonging of user *k*
*Y*_*k*_	Labels for sample data	*y*	Label category
*C*	User’s cluster	*T*	Global operations
*t*	User’s *t*th round	*θ* ^0^	Initial global model

## The Fed-GANCC framework

### Framework overview

The Fed-GANCC framework comprises three components: (1) the generative adversarial model at the central server; (2) the AP node’s generative user model feature classification layer; (3) the user node, responsible for user behavior data collection, data enhancement, and personalized grouping aggregation.

- The generative adversarial model creates data similar to the training set by generating samples z ∼ *p*_*z*_(x) from training data g ∼ *p*_*g*_(x). The adversarial model generates a public dataset, evaluated by the DMM distance metric between the generated and training datasets.
- A conditional generative adversarial network can be used to balance local user data category distribution by augmenting missing data, thereby improving data quality.
- The central server performs personalized grouping aggregation. However, in targeted advertising, the FedAvg algorithm may suffer from bias and reduced accuracy due to the non-independent and non-identically distributed user behavior data.

### Generative Adversarial Network scheme

#### Motivation for the Generative Adversarial Network

- Non-IID data causes inefficiencies in federated learning algorithms. This divergence in the FedAvg algorithm results from biased data distribution estimation by user nodes [28].
- When using GANs, we opted for a third-party dataset scheme for better privacy, despite potential local ML parameter inconsistencies. We enhanced this scheme by managing GAN models centrally and performing uniform adjustments for stable, high-quality data generation [29].
- Traditional GANs use the Inception Score (IS) as an evaluation index, which has certain limitations. Instead, we use Maximum Mean Discrepancy (MMD) to measure training and generated dataset distances. Lower MMD values indicate better GAN performance.

To tackle these, data can be augmented to alleviate user nodes’ data distribution discrepancies. This method relies on data augmentation principles and is implemented by adding extra data to the server through GANs, bypassing user node access limitations. The validity of this approach will be further explored in the experimental section.

#### The GAN-based procedure

**Fig 3:**
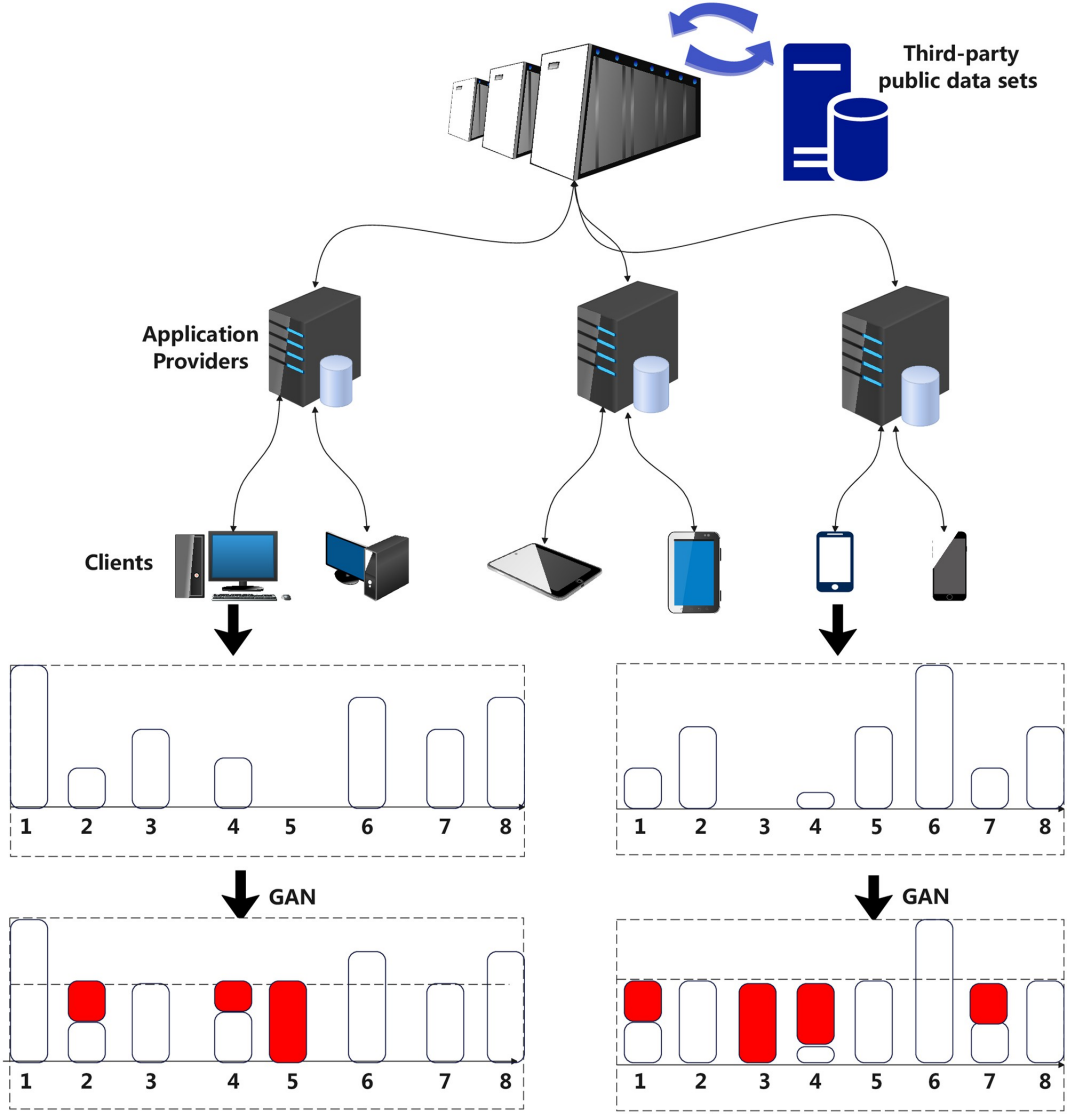
Generative Adversarial Network-based framework diagram for user behavior data augmentation.

Fig 3 portrays the user behavior data augmentation process using Generative Adversarial Networks (GANs). The central server first dispatches a GAN model to the Clients. This model, grounded on a third-party public dataset, is designed to mitigate the non-IID nature of the user behavior data stored in the clients. Using this dispatched GAN model, Clients then create synthetic data, which enriches their local user behavior data.

In this process, the mathematical model of adversarial learning takes on the form of a zero-sum game. The players of this game are the Discriminator function DN(X): *R*^*n*^ → [0, 1] and the generator’s *Gz* function: *R*^*d*^ → *R*^*n*^. The generator creates sample *Gz* from random sample *z* following the *Rn* distribution *o*, intending to mimic the distribution of the training samples. In contrast, the discriminator DN aspires to separate these synthetic samples from the training samples drawn from distribution *c*. This adversarial dynamic is captured by the following loss function:

$$\begin{array}{c} \begin{array}{c} \hat{V}\left(DN,G_{z}\right)≔\\ Ex∼c[\text{log}\;DN(x)]+Ew∼o[\text{log}\;(1-DN(G(w)))] \end{array} \end{array}$$ (1)

The optimal equilibrium for GAN can be represented as:

$$\begin{array}{c} \begin{array}{c} \underset{G_{z}}{\text{min}}\text{max}DN\hat{V}\left(DN,G_{z}\right)≔\\ \underset{G_{z}}{min}\;\underset{DN}{max}(Ex∼c\left[\text{log}\;DN\left(x\right)\right]+E_{w∼o}\left[\text{log}\;\left(1-DN\left(G_{z}\left(w\right)\right)\right)\right]) \end{array} \end{array}$$ (2)

Rewriting the above equation in terms of variables *DN*, *G*_*z*_, and the newly introduced *Y* = *G*_*z*_(z)∈*R*^*n*^, which follows distribution $v≔o\cdot G_{z}^{-1}$, we get:

$$\begin{array}{c} \begin{array}{cc} \tilde{V}\left(DN,v\right)≔ & \hat{V}\left(DN,G_{z}\right)\\ & =E_{x∼c}\left[\text{log}\;DN\left(x\right)\right]+E_{w∼o}\left[\text{log}\;\left(1-DN\left(G_{z}\left(w\right)\right)\right)\right]\\ & =E_{x∼c}\left[\text{log}\;DN\left(x\right)\right]+E_{Y∼o}\left[\text{log}\;\left(1-DN\left(Y\right)\right)\right]\\ & =\int_{R^{n}}\text{log}\;DN\left(x\right)dc\left(x\right)+\int_{R^{n}}\text{log}\;\left(1-DN\left(Y\right)\right)dv\left(Y\right) \end{array} \end{array}$$ (3)

This max-min problem is then recast into:

$$\begin{array}{c} \begin{array}{c} \text{min}G_{z}\text{max}DN\hat{V}\left(DN,G_{z}\right)≔\\ \underset{G_{Z}}{min}\;\underset{DN}{max}(\int R^{n}\text{log}\;DN\left(x\right)dc\left(x\right)+\int R^{n}\text{log}\;\left(1-DN\left(Y\right)\right)dv\left(Y\right)) \end{array} \end{array}$$ (4)

Our aim is to construct a model that closely matches the actual data distribution. We quantify this similarity using the Maximum Mean Discrepancy (MMD) distance, computed using a Gaussian kernel function *K*_*xy*_(*x*, *y*):

$$\begin{array}{c} K_{xy}\left(x,y\right)=ep({-|x-y|}^{2}) \end{array}$$ (5)

The MMD distance is then:

$$\begin{array}{c} \begin{array}{c} E_{x,x^{'}∼p_{\text{dota}\;}}[k_{xy}(x,x^{'})]-2E_{x∼p_{\text{data}\;},Y},\\ Yp_{g}\left[k_{xy}\left(x,y\right)\right]+E_{y,y^{'}∼p_{g}}[k(y,y^{'})] \end{array} \end{array}$$ (6)

The true expectation is computationally intractable, thus we resort to sample estimation for the MMD value. Given n samples *x*_1_, …, *x*_*n*_ from the training set and n samples *y*_1_, …, *y*_*n*_ generated by *G*_*z*_, the estimated MMD is:

$$\begin{array}{c} \frac{1}{C_{n}^{2}}\sum_{i\neq i}k_{xy}(x_{i},x_{i}^{'})-\frac{2}{C_{n}^{2}}\sum_{i\neq j}k_{xy}(x_{i},y_{j})+\frac{1}{C_{n}^{2}}\sum_{j\neq j}k_{xy}(y_{j},y_{j}^{'}) \end{array}$$ (7)

Given the unavailability of the true data distribution Pdata(x), we approximate it using the empirical distribution of the training data. This allows the generated samples to exhibit a feature distribution similar to the training samples.

### Clustering-based classification layer scheme

#### Motivation for classification layer clustering

Federated Learning (FL) embodies an innovative distributed learning method protecting user data privacy by leveraging local Machine Learning (ML) parameters without direct user data access. Nonetheless, FL’s effectiveness diminishes in real-world scenarios such as targeted advertisement recommendations, where non-IID data presents significant challenges. The inherent limitation of FL approaches lies in their inability to account for disparities in user data distributions while updating a single global model by aggregating user-specific gradients.

We address this issue through the implementation of a cluster-based federated learning approach, drawing on the success of such methods in combating concept drift. This strategy entails the allocation of users to different global models (or centers), enabling the amalgamation of multiple global models to capture non-IID data across diverse users.

Yet, the conventional clustering methods that anchor their strategies on the test accuracy of user models fall short in non-IID scenarios like targeted advertisement recommendation. These methods often group high accuracy models with extreme models, specifically those only equipped to perform certain types of operations, which clearly diverges from the optimal targeted ad delivery objective [30, 31].

To address these shortcomings, we propose a novel approach that employs classification layer similarities as the clustering basis. This methodology facilitates the effective grouping of users with similar interests, consequently reducing model training time. The validity and effectiveness of this approach will be demonstrated in subsequent experimental sections.

#### Classification layer clustering-based procedure

This paper introduces a two-stage personalized aggregation and grouping strategy that leverages classification layer similarities, eschewing the conventional practice of using the model’s test accuracy as the clustering foundation. The method initiates with users training their models utilizing improved user behavior data, which is subsequently uploaded to the Access Point (AP) node via the central server. The server executes the Federated Averaging (FedAvg) algorithm first, followed by feature extraction layer aggregation.

$$\begin{array}{c} \theta^{k}=\sum_{i=1}^{I}\frac{pi}{\sum {k=1}^{I}pi}\theta {t+1}^{i} \end{array}$$ (8)

Here, *θ*_*t*+1_ represents the global model parameters for the (t+1) round, and *p*_*i*_ symbolizes the i-th user node contributing to the computation. $\theta_{t+1}^{i}$ are the user model parameters included in the calculation, while $\sum_{i=1}^{I}$ denotes the total user count.

The local model’s classification layer serves as the clustering subject, with the server running the clustering algorithm to yield user grouping outcomes. Here, $w_{i}^{*}$ is the i-th user group’s optimal cluster model, and $r_{k}^{\left(i\right)}$ denotes user k’s cluster membership (if n∈*S*_*i*_, then $r_{k}^{\left(i\right)}$ = 1; otherwise, $r_{k}^{\left(i\right)}$ = 0). The User symbolizes the entire user set, argmin *f*_*k*_(x) indicates the variable value corresponding to the minimum of the objective function *f*_*k*_(x), and C represents the number of groups.

$$\begin{array}{c} \left[wi^{*}]≜\underset{wi}{\text{argmin}}\sum n\in \;\text{User}\;\sum {i=1}^{C}r_{k}^{\left(i\right)}f_{k}(w_{i}\right) \end{array}$$ (9)

The classification layer undergoes grouping and aggregation to produce various cluster models, which are subsequently disseminated to the corresponding user groups. Here, argmin *f*_*n*_(x) signifies the variable value where the objective function *f*_*n*_(x) for user n is minimal, and *S*_*i*_ denotes the i-th user group. The optimal cluster model for the i-th user group is represented by $w_{i}^{*}$.

$$\begin{array}{c} w_{i}^{*}=\underset{n\in S_{i}}{\text{argmin}}f_{n}(w_{i}) \end{array}$$ (10)

### Pseudocode of Fed-GANCC framework

**Algorithm 1** User Behavior Data Enhancement Based on Generative Adversarial Network

**Require**: The generated adversarial network model *G*

1: **for** *i* = 1 to *N* **do**

2:  $Z\leftarrow \text{max}|Y_{i}^{y}|$

3:  **for** each *y* in $Y_{i}^{y}$ **do**

4:   **if** $|Y_{i}^{y}|<\alpha \times Z$ **then**

5:    Use the model *G* to expand the data volume of the category data to *α* × *Z*

6:   **end if**

7:  **end for**

8: **end for**

This section provides the pseudo-code of the Fed-GANCC framework, including the user behavior data enhancement module (see Algorithm 1) and the personalized grouping aggregation module node update part (see Algorithm 2).

Algorithm 1: where $|Y_{i}^{y}|$ represents the number of data labeled with y in the i-th user. The user behavior data enhancement part takes the adversarial network model G generated by Sever as input. Line 4 represents the calculation of the maximum number of label classes; line 5 represents the user traversing all the data label classes in the local; line 7-9 represent data enhancement for the data classes that need to be expanded respectively.

Algorithm 2 Personalized grouping aggregation module update part takes T, P, t = 0, and *θ*^0^ are the inputs for the algorithm 2’s personalized grouping aggregation module update component, where T denotes the number of global iterations, P, the total number of users, t, and *θ*^0^, the initial global model. Set B to reflect the data batch size for the vehicle node. Specifically, line 5-8 represents the local model training of each user and the upload of the local model; line 10 represents the running of the FedAvg algorithm and the aggregation of the feature extraction layer; line 9 represents the obtaining of the latest dataset; line 11-15 represents the taking of the classification layer of the local model as the clustering object, the running of the clustering algorithm to produce the user grouping results; the grouping and aggregation of the classification layer to generate a number of cluster models, which are then distributed to the corresponding user groups; The computation of the global model parameters and the associated performance metrics ACC, Loss, FPR, and TPR are shown on lines 16 through 25, and their return to the AP is shown on line 27.

**Algorithm 2** Personalized Grouping Aggregation Based on Classification Layer

**Require**: Global iteration times *T*, *P*, *t* = 0, initial global model *θ*^0^ = (*g*^0^, *h*^0^)

1: **While** *t* < *T* **do**

  {User} **for** each *p*_*i*_ in *P* **do**

3:  $w_{i}^{t}=\underset{w}{\text{argmin}}f_{n}(w)$

4:  Upload $w_{i}^{t}≜\left(g_{n}^{t},h_{n}^{t}\right)$ and sample number *S*

5:  **end for** {Server}

6:  $\theta^{k}=\sum_{i=1}^{I}\frac{p_{i}}{\sum_{k=1}^{I}p_{i}}\theta_{t+1}^{i}$

7:  Run the clustering algorithm on the classification layer to get each user cluster $r_{k}^{(c)t}$

8:  $w^{(q)t}≜\underset{w_{i}}{\text{argmin}}\sum_{k\in \{User\}}\sum_{i=1}^{c}r_{k}^{\left(i\right)}f_{k}(w_{i})$

9:  **w**^(q)t^ = (g^t^, h^(q)t^), q ∈ 1, 2, 3, …, q

10:  $h^{(q)t}=\sum_{k\in [1,N]}r_{k}^{(q)t}\cdot \frac{D_{n}}{\sum_{k\in [1,N]}r_{k}^{(q)t}\cdot D_{k}}\cdot h_{k}^{t}$

11:  According to $r_{k}^{(q)t}$ send the cluster model ***w***^(c)t^ to the corresponding user

12:  **for** each local epoch *t* from 1 to *T* **do**

13:   batches ← (data *D*_*i*_ split into batches of size *B*)

14:   **for** each batch *b* in batches **do**

15:    $Max\_\text{Accuracy}=\frac{1}{\;N}\sum_{i=1}^{N}\;\text{softmax}(w_{y}\cdot \Gamma_{\text{out}\;}^{\left(t\right)}\cdot \text{tanh}(c^{\left(t\right)})+b)/Y_{t}$

16:    $Min\_FPR=\frac{FP}{FP+TN}$

17:    $Max\_TPR=\frac{TP}{TP+FN}$

18:    $\theta_{t+1}\leftarrow \sum_{i=1}^{I}\frac{p_{i}}{p}\theta_{t+1}^{i}$

19:   **end for**

20:  **end for**

21:  *t* = *t* + 1

22:  **return** ACC, Loss, FPR, TPR, *θ*

23: **end while**

## Mathematical proof of the Fed-GANCC framework

### Formal definition and property analysis of Generative Adversarial Networks (GANs)

#### Strict definition and basic properties of Generative Adversarial Networks

**Definition 1** *First, let us formally define Generative Adversarial Networks (GANs) from the perspective of deep learning. In a GAN, we have two key components: a generator G*, *whose objective is to generate data samples that are as realistic as possible, and a discriminator D*, *whose objective is to accurately distinguish between real data and data generated by the generator. Together, they form a dynamic “zero-sum game” framework. We can describe this process using the following min-max problem*:

$$\begin{array}{c} \begin{array}{c} \underset{G}{\text{min}}\;\underset{D}{\text{max}}V\left(D,G\right)=\\ Ex∼pdata(x)[\text{log}\;D(x)]+Ez∼pz(z)[\text{log}\;(1-D(G(z)))] \end{array} \end{array}$$ (11)

Here, ***x*** represents real data, ***z*** represents noise sampled from a prior distribution, *p*_data_ is the distribution of real data, and *p*_**z**_ is the distribution of noise. In this process, the generator *G* and the discriminator *D* are optimized alternately until a Nash equilibrium is reached.

#### Enhancing user behavior data with Generative Adversarial Networks

In this section, we provide a detailed description of how to apply generative adversarial networks to enhance user behavior data. We take user behavior data as real data and optimize the GAN framework so that the generator *G* can generate data with similar statistical properties to real user behavior data, thereby achieving data augmentation.

**Definition 2** *When enhancing user behavior data, we can define a loss function L_user_ based on the distribution difference between the generated data and the real data, aiming to minimize this difference*:

$$\begin{array}{c} L_{user}=D_{KL}(p_{data}\left(x\right)||p_{G}\left(G\left(z\right)\right)) \end{array}$$ (12)

Here, *D*_*KL*_ denotes the Kullback-Leibler divergence, which measures the difference between two probability distributions. By continuously optimizing this loss function, we can make the generated data increasingly close to real user behavior data.

### Formal definition and property analysis of group clustering algorithm

#### Strict definition and basic properties of group clustering algorithm

In this section, we formally define the Group Clustering algorithm and discuss its basic properties. Group Clustering is a specific type of clustering algorithm that aims to partition data points into different groups (or clusters) under certain constraints. These constraints are often determined by our understanding of the data and the requirements of the task.

**Definition 3** *Given a dataset D* = *x*_1_, *x*_2_, …, *x*_*n*_, *Group Clustering can be seen as an optimization problem*:

$$\begin{array}{c} \underset{G}{\text{min}}\sum_{i=1}^{k}\sum_{x_{j}\in G_{i}}d\left(x_{j},c_{i}\right) \end{array}$$ (13)

Here, *G* = *G*_1_, *G*_2_, …, *G*_*k*_ represents the partition of clusters, *c*_*i*_ is the centroid of the *i*-th cluster, and *d*(*x*_*j*_, *c*_*i*_) is a distance metric (e.g., Euclidean distance) that measures the distance between data point *x*_*j*_ and cluster center *c*_*i*_. Our goal is to find a partition *G* that minimizes this objective function.

#### Group clustering with data generated by Generative Adversarial Networks (GANs)

In this section, we will elaborate on how to perform Group Clustering using data generated by Generative Adversarial Networks (GANs). By generating data through GANs, we can obtain richer and more diverse user behavior patterns, which helps us classify users more accurately.

**Definition 4** *Assuming we have generated a set of data* $D^{'}=x_{1}^{'},x_{2}^{'},\ldots ,x_{m}^{'}$ *using GANs, our Group Clustering problem can be extended to the following optimization problem*:

$$\begin{array}{c} \underset{G}{\text{min}}\sum_{i=1}^{k}\left(\sum_{x_{j}\in G_{i}}d\left(x_{j},c_{i}\right)+\sum_{x_{j}^{'}\in G_{i}}d\left(x_{j}^{'},c_{i}\right)\right) \end{array}$$ (14)

### Properties of parameter solution set satisfying Generative Adversarial Networks (GANs) and group clustering conditions

#### Theorem: Existence of parameter solution set

**Theorem 1** *For the parameter space* Θ *that satisfies the defined conditions of Generative Adversarial Networks (GANs) and Group Clustering, there exists a non-empty open set whose closure is a convex set satisfying the constraints, denoted as* Θ′.

[Proof of Theorem 1] In order to prove the theorem, we start by building a mathematical model for the parameter solutions of GANs and Group Clustering algorithms. First, we define the parameter vector *θ* = (*θ*_1_, *θ*_2_, …, *θ*_*p*_) and construct the following optimization problem:

$$\begin{array}{c} \underset{\theta }{\text{min}}\;L\left(D,G,\theta \right)\;\text{s.t.}\;C\left(\theta \right)\leq 0 \end{array}$$ (15)

Here, *L*(*D*, *G*, *θ*) denotes the loss function, *D* is the original dataset, *G* refers to the dataset generated by GANs, and *C*(*θ*)≤0 represents the constraints for GANs and the Group Clustering algorithm.

**Assumption 1:** The loss function *L*(*D*, *G*, *θ*) is continuous and bounded within the domain of the parameter solutions. Let’s further break down this assumption:

$$\begin{array}{c} \forall \epsilon >0,\exists \delta >0\;s.t.\;|L(D,G,\theta +\delta )-L(D,G,\theta )|<\epsilon \end{array}$$ (16)

**Assumption 2:** The constraint *C*(*θ*)≤0 defines a closed feasible region. In mathematical terms:

$$\begin{array}{c} \forall \theta_{n}\in \Theta ,\;\theta_{n}\to \theta \;implies\;C\left(\theta \right)\leq 0 \end{array}$$ (17)

**Assumption 3:** The parameter space Θ is a complete metric space:

$$\begin{array}{c} \forall {\left\{\theta_{n}\right\}}_{n=1}^{\infty }\subset \Theta ,\;\theta_{n}\to \theta \;implies\;\theta \in \Theta \end{array}$$ (18)

According to the Baire category theorem, the parameter space Θ must contain a non-empty open set, whose closure is a convex set. Let’s denote this set as Θ′.

$$\begin{array}{c} \Theta^{'}\subset \Theta ,\;\Theta^{'}\;\text{is}\;\text{convex} \end{array}$$ (19)

We then need to prove that within Θ′, there exists θ* ∈ Θ′ such that *L*(*D*, *G*, *θ*) ≤ *L*(*D*, *G*, *θ*) for all *θ* ∈ Θ′ satisfying *C*(*θ*)≤0. Therefore, θ* is the solution set that satisfies the defined conditions of GANs and Group Clustering.

$$\begin{array}{c} \exists \;\theta^{*}\in \Theta^{'},\;\forall \theta \in \Theta^{'},\;C\left(\theta \right)\leq 0\;implies\;L\left(D,G,\theta^{*}\right)\leq L\left(D,G,\theta \right) \end{array}$$ (20)

This completes the proof of the existence of a parameter solution set that satisfies the conditions defined for GANs and Group Clustering. Further analysis on uniqueness and stability of solutions will require more in-depth research.

#### Theorem: Boundedness of parameter solution set

**Theorem 2** *For the parameter space* Θ′ *that satisfies the defined conditions of Generative Adversarial Networks (GANs) and Group Clustering, we can find a finite constant M* > 0 *such that for any θ* ∈ Θ′, *we have* |*θ*| ≤ *M*.

[Proof of Theorem 2] We set a new set of assumptions, shifting the focus of the proof to the loss function *L*(*D*, *G*, *θ*) and the function $F:\Theta^{'}\to R$.

**Assumption 4:** The loss function *L*(*D*, *G*, *θ*) is continuous and bounded, meaning that there exists a finite constant *L* > 0 such that *L*(*D*, *G*, *θ*)≤*L* holds for all *θ* ∈ Θ′.

**Assumption 5:** There exists a function $F:\Theta^{'}\to R$ such that *F*(*θ*) = *L*(*D*, *G*, *θ*), and *F* is continuous and bounded on Θ′.

Next, we proceed by contradiction. Suppose Θ′ is unbounded, then there exists a sequence ${\left(\theta_{n}\right)}_{n=1}^{\infty }\subset \Theta^{'}$ such that |*θ*_*n*_|→∞ as *n* → ∞.

Based on Assumption 5, we know that *F*(*θ*_*n*_) is bounded, which means there exists a constant *F* > 0 such that |*F*(*θ*_*n*_)| ≤ *F* holds for all *n*. According to the *Heine-Borel Theorem*, if a function *F* is bounded, then its domain must be bounded.

Therefore, by Assumption 5, we can find a sequence ${\left(x_{n}\right)}_{n=1}^{\infty }$ such that |*x*_*n*_|→∞ as *n* → ∞, and *F*(*x*_*n*_) is bounded. This contradicts Assumption 4 because if |*x*_*n*_|→∞, then according to Assumption 4, we cannot find a constant *L* > 0 such that *L*(*D*, *G*, *x*_*n*_)≤*L* holds for all *n*.

Thus, we can conclude that the parameter space Θ′ that satisfies the defined conditions of GANs and Group Clustering must be bounded. This completes the proof of the boundedness of the parameter solution set.

#### Theorem: Convergence of parameter solution set

**Theorem 3** *In the parameter space* Θ′ *which fulfills the conditions of the Generative Adversarial Networks (GANs) and Group Clustering, a sequence* ${\left(\theta_{n}\right)}_{n=1}^{\infty }\subset \Theta^{'}$ *can be constructed such that* |*θ*_*n*_ − *θ*| → 0 as *n* → ∞. *Here, θ is a parameter solution complying with our conditions*.

[Proof of Theorem 3] Continuing with our earlier defined loss function *L*(*D*, *G*, *θ*) and function $F:\Theta^{'}\to R$, we introduce new mathematical tools for the proof.

**Assumption 6:** There exists a function $F:\Theta^{'}\to R$ such that *F*(*θ*) = *L*(*D*, *G*, *θ*), and *F* exhibits continuity over Θ′.

**Assumption 7:** A sequence ${\left(\theta_{n}\right)}_{n=1}^{\infty }\subset \Theta^{'}$ can be found such that |*θ*_*n*_ − *θ*| → 0 as *n* → ∞, where *θ* is a parameter solution fulfilling our conditions.

In the first part of the proof, we aim to demonstrate the continuity of *F* over Θ′. For any *ϵ* > 0, there exists *δ* > 0 such that when |*θ*′ − *θ*| < *δ*, it leads to |*F*(*θ*′) − *F*(*θ*)| < *ϵ*. This completes the proof of *F*’s continuity on Θ′.

$$\begin{array}{c} \forall \epsilon >0,\exists \delta >0,|\theta^{'}-\theta |<\delta \Rightarrow |F\left(\theta^{'}\right)-F\left(\theta \right)|<\epsilon \end{array}$$ (21)

Next, we demonstrate the convergence of the parameter solution set. As per the definition of function continuity, there exists a sequence ${\left(\theta_{n}\right)}_{n=1}^{\infty }\subset \Theta^{'}$ such that |*θ*_*n*_ − *θ*| → 0 as *n* → ∞, thus ${\left(\theta_{n}\right)}_{n=1}^{\infty }$ converges to *θ*.

$$\begin{array}{c} \forall \epsilon >0,\exists N\in N,n>N\Rightarrow |\theta_{n}-\theta |<\epsilon \end{array}$$ (22)

Lastly, we need to show that the sequence ${\left(F\left(\theta_{n}\right)\right)}_{n=1}^{\infty }$ also converges to *F*(*θ*). For any *ϵ* > 0, as *F* is continuous on Θ′, there exists N∈N such that for all *n* > *N*, we have |*F*(*θ*_*n*_) − *F*(*θ*)| < *ϵ*. Hence, the sequence ${\left(F\left(\theta_{n}\right)\right)}_{n=1}^{\infty }$ converges to *F*(*θ*).

$$\begin{array}{c} \forall \epsilon >0,\exists N\in N,n>N\Rightarrow |F\left(\theta_{n}\right)-F\left(\theta \right)|<\epsilon \end{array}$$ (23)

This concludes the proof.

#### Corollary: Closure property of parameter solution set satisfying GANs and group clustering conditions

**Corollary 1** *Let* Θ′ *be a parameter solution set that satisfies the existence* (*P*_*e*_), *boundedness* (*P*_*b*_), *and convergence* (*P*_*c*_) *properties as proven under the conditions of GANs and Group Clustering. Then, the closure* $\bar{\Theta^{'}}$ of the parameter solution set Θ′ will also possess these properties.

The importance of this corollary in the paper lies in the fact that the existence, boundedness, and convergence properties of the parameter solution set are crucial in the parameter optimization process when using the federated learning framework for targeted advertising. This corollary further ensures that these properties are maintained in the closure of the parameter solution set, providing theoretical guarantees for finding global optimal solutions in a distributed setting.

[Proof of Corollary 1] Given that we have already proven the properties of the parameter solution set Θ′ under conditions *P*_*e*_, *P*_*b*_, and *P*_*c*_, we now aim to show that these properties also hold for the closure $\bar{\Theta^{'}}$ of Θ′.

Existence: Since Θ′ is non-empty, by the definition of closure, we have $\bar{\Theta^{'}}\neq \emptyset$. Boundedness: As Θ′ is bounded, there exists *R* > 0 such that for all *θ* ∈ Θ′, we have |*θ*|≤*R*. According to the properties of closure, for any $\bar{\theta }\in \bar{\Theta^{'}}$, there exists a sequence of *θ* converging to $\bar{\theta }$ within Θ′. Therefore, we have $|\bar{\theta }|\leq R$, implying that $\bar{\Theta^{'}}$ is bounded. Convergence: Considering any convergent sequence of Θ′, its limit point must belong to $\bar{\Theta^{'}}$. Thus, any limit point of a convergent sequence of Θ′ also belongs to $\bar{\Theta^{'}}$, proving the convergence property of $\bar{\Theta^{'}}$. Thus, we have shown that if the parameter solution set Θ′ satisfies *P*_*e*_, *P*_*b*_, and *P*_*c*_, then the closure $\bar{\Theta^{'}}$ of Θ′ will also possess these properties.

## Experimental result

### Dataset description

We employed two click-through rate prediction datasets, Ali_Display_Ad_Click (ADAC) and Click-Through Rate Data (CTRD), respectively sourced from open data platform Ali Tianchi1 and Kaggle Online Advertising Click-Through Rate Prediction Competition2. ADAC dataset contains 20 fields and 704,319 data points, of which 80% was used as the training dataset and the remaining 20% as the test dataset. Kun Gai et al. proposed a Large Scale Piece-wise Linear Model (LS-PLM) for click-through rate (CTR) prediction in real-world business, and verified it with the ADAC dataset [32]. Guorui Zhou et al. proposed a Deep Interest Network (DIN) for online advertising click-through rate prediction, and used the same dataset to validate the model [33]. CTRD dataset consists of 17 fields and 80,000 training data points and 20,000 test data points. Both datasets are used for online advertising click-through rate prediction, and can effectively reflect whether the advertisement is effectively delivered to the specified people, thereby verifying the ability of Fed-GANCC model. Therefore, we selected these two datasets as the experiment dataset.

### Experimental parameter settings

The specific configuration of the experimental platform is: 12th Gen Intel(R) Core(TM) i7-12700H CPU@ 2.30GHz, DDR4 2933MHz 16GB memory, Windows 11 Home Edition 64-bit operating system, The experimental software uses python 3.6, pytorch 1.4. 0+cpu and Keras 2.4.3.

This experiment uses LSTM to verify the advertising click-through rate dataset. The LSTM framework structure is an input layer, a hidden layer, and an output layer. The specific experimental parameter settings are shown in Table 2.

**Table 2:** Experimental parameter table.

Parameter Category	Parameter Name	ADAC And CTRD
LSTM Parameters	Number of data vector dimensions	17
	hidden meta dimension	128
	Number of LSTM layers connected	3
	Linear layer 1	128*64
	Linear layer 2	64*32
	Linear layer 3	32*14
	batch_first	True
	dropout	0.2
	bidirectional	True
Federated Learning Parameters	Global training rounds	10
	Local training rounds	5
	Initial advertisements	10
	Data Distribution	non-IID
	Learning rate	0.01
	Decay rate	0.1
	Processor	Cpu

### Experimental results

- Accuracy Results

**Fig 4:**
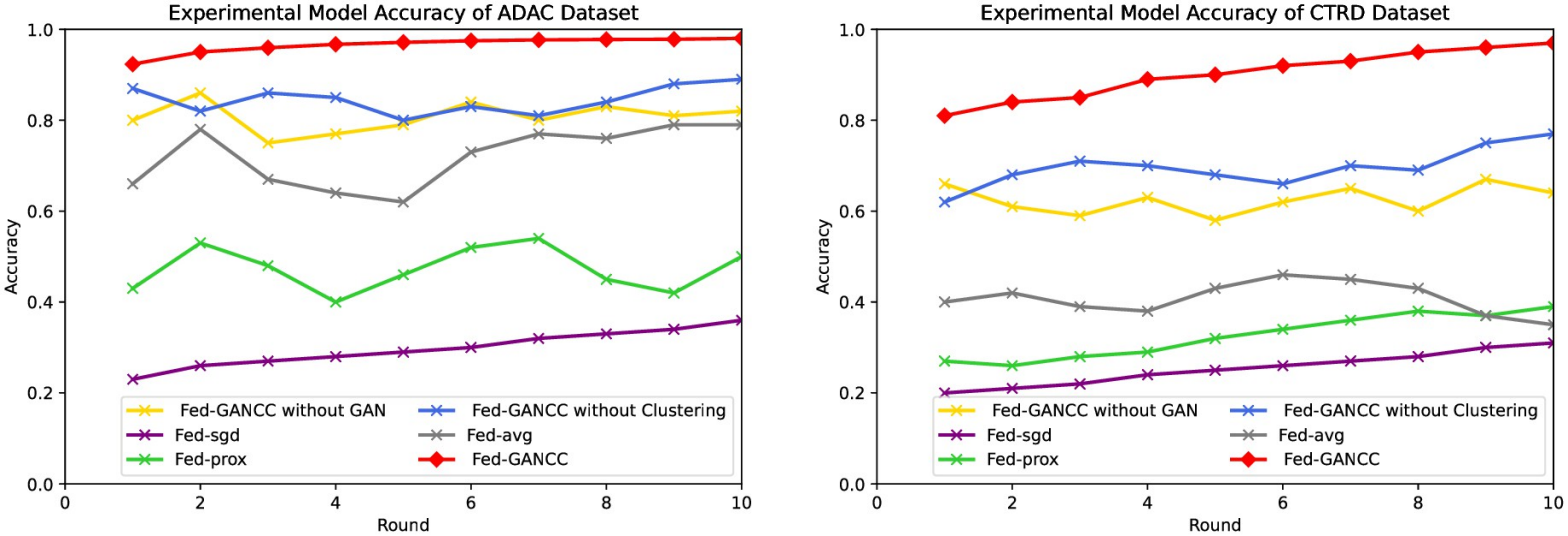
Comparison of accuracy.

We compared the Acc, Loss, and ROC curves of the Fed-AVG, Fed-SGD, Fed-prox, and Fed-GANCC. As evidenced in Fig 4, only Fed-Sgd and Fed-GANCC have their precision steadily increasing with the number of global ML model rounds across all frameworks, with the enhancement of Fed-sdg being negligible. Moreover, the Fed-GANCC model exhibits an accuracy that is approximately 30% higher than the Fed-avg model on the ADAC dataset. When using the CTRD dataset, this difference increased to 40%. Furthermore, the Fed-prox framework was observed to be highly unstable during the experiment, and the Fed-sgd framework performed poorly. We believe this is mainly due to the strong tendency that the user uploaded local models have, resulting in both models failing to adapt.

- Loss Results

**Fig 5:**
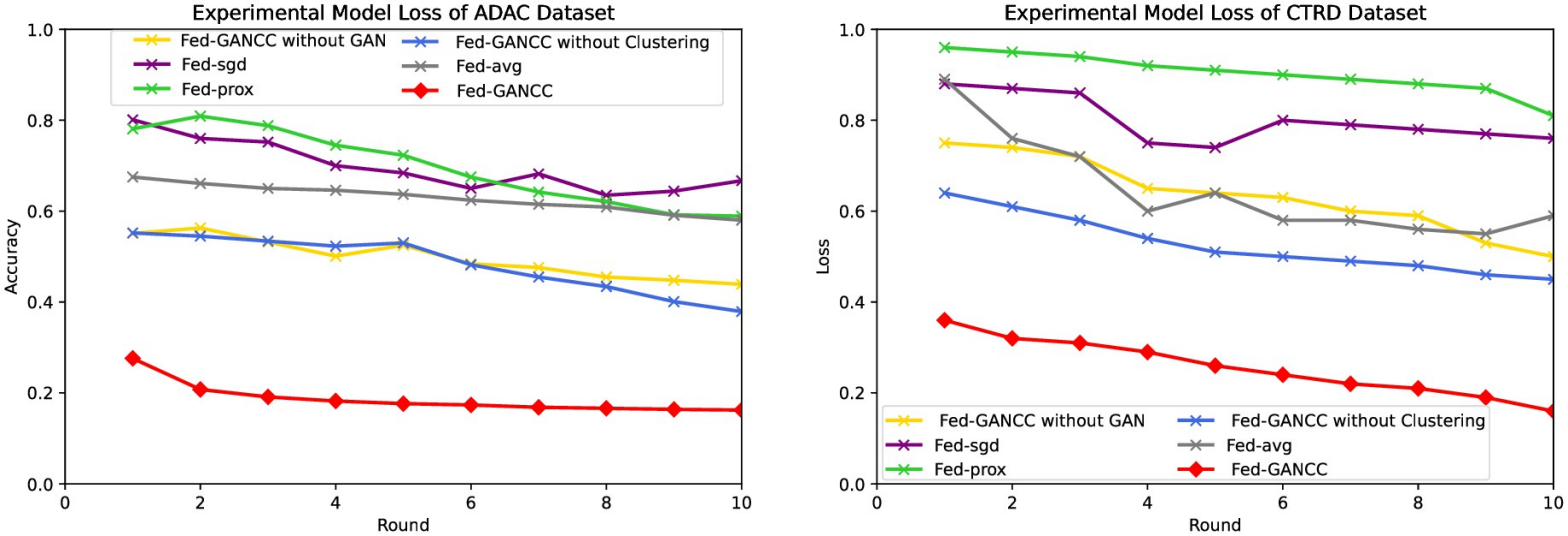
Comparison of loss.

Fig 5 provides insights into the effect of global ML model training cycles on the stability of different federated learning frameworks. Specifically, as shown in the figure, the loss value of Fed-AVG tends to become increasingly unstable as the number of cycles increases across both datasets. Out of the four frameworks considered, only Fed-GANCC and Fed-prox exhibit relative stability, albeit with a noticeable gap between the two.

- ROC Results

**Fig 6:**
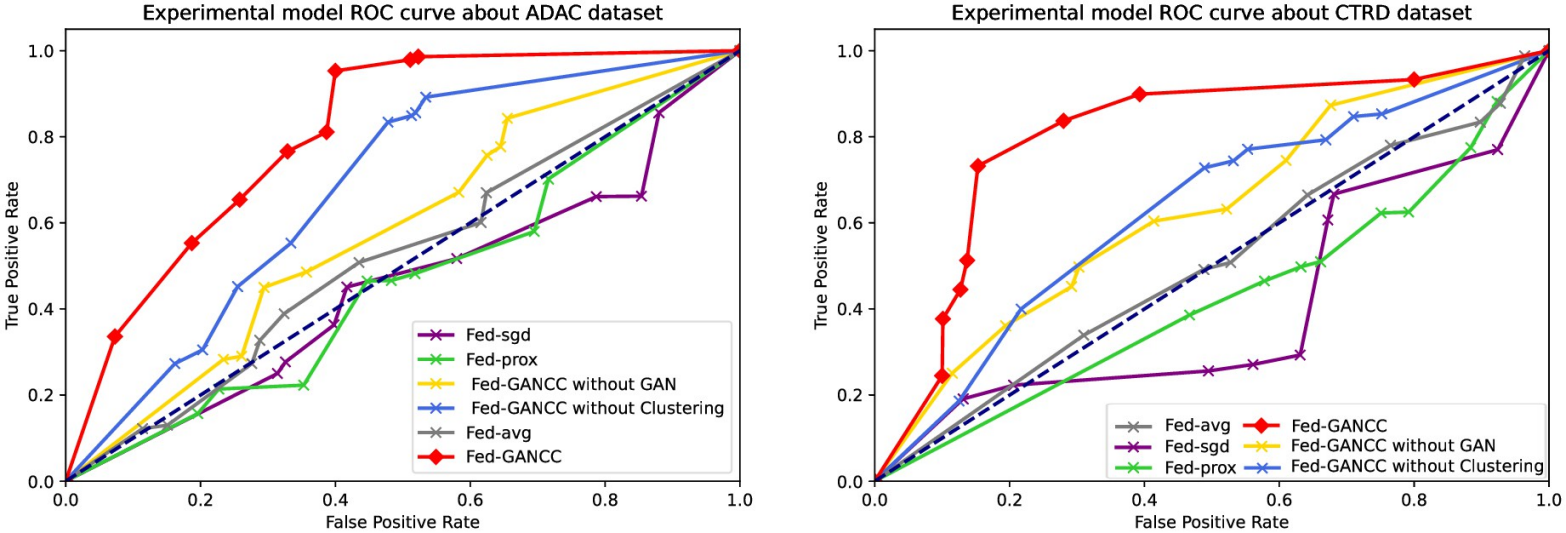
Comparison of ROC.

We compared the ROC of the above six frameworks as shown in Fig 6. Fed-sgd and Fed-prox were both below the dashed line in both datasets, indicating that their performance was lower than random prediction and had low learning efficiency. Based on the comparison of these frameworks, only FED-AVG performed well, but their performance was still much weaker than the Fed-GANCC framework.

Regardless of whether the ADAC or CTRD dataset is used, the Fed-GANCC framework is able to maintain a high level of user prediction, outperforming several common federated learning frameworks such as FED-AVG, FED-SGD, and FED-prox.

### Discussion

Despite the promising results demonstrated by Fed-GANCC, it’s essential to address its limitations. For instance, while the Generative Adversarial Network scheme is proficient at addressing data distribution disparities, it may require substantial computational resources, potentially limiting its application in resource-constrained environments. Additionally, the Classification Layer Clustering might not be universally applicable for all types of data drift scenarios, and further studies would be required to tailor it for specific applications. Regarding the applicability of Fed-GANCC, while it excels in environments where data distribution is heterogeneous and subject to drift, it might not be the most suitable for homogeneous data distribution settings or scenarios where computational resources are sparse. However, in domains like targeted advertising where user behavior is dynamic and evolves over time, Fed-GANCC stands as a potent tool.

## Conclusion

The challenges posed non-IID and concept drift in targeted advertising have resulted in poor performance of traditional federated learning frameworks. To mitigate these challenges, this paper introduces a novel federated learning framework, named Fed-GANCC, which combines the principles of Generative Adversarial Networks and Classification Layer Clustering. To address the data distribution gap between user nodes, the framework employs a Generative Adversarial Network scheme. Additionally, the framework leverages a Classification Layer Clustering scheme to handle the adversarial effects of concept drift in the targeted advertising domain. An evaluation of the Fed-GANCC framework was conducted using a benchmark dataset. The comparison of the performance of Fed-GANCC with existing baseline frameworks revealed that the proposed framework achieved better results than the baseline approaches, as evidenced by metrics such as Accuracy, Loss, and ROC curve. Our findings demonstrate the potential of Fed-GANCC in addressing the issues of isolated data islands, non-IID, and concept drift in targeted advertising and make a substantial contribution to the area of federated learning.

Our study has paved the way for further research in enhancing federated learning frameworks for advertising. Possible future research directions include: 1)Exploring variations of the Generative Adversarial Network scheme tailored for different types of non-IID data. 2) Investigating the scalability of Fed-GANCC for larger and more diverse datasets. 3) Designing robustness mechanisms against adversarial attacks in the context of federated learning. In conclusion, our findings not only highlight the potential of Fed-GANCC in addressing the challenges in targeted advertising but also provide a balanced view of its strengths and areas for future enhancements, thereby solidifying its position as a noteworthy contribution to federated learning.
